# Can Plant Defence Mechanisms Provide New Approaches for the Sustainable Control of the Two-Spotted Spider Mite *Tetranychus urticae*?

**DOI:** 10.3390/ijms19020614

**Published:** 2018-02-21

**Authors:** Blas Agut, Victoria Pastor, Josep A. Jaques, Victor Flors

**Affiliations:** 1 Departament de Ciències Agràries i del Medi Natural. Campus del Riu Sec, Metabolic Integration and Cell Signalling Group, Universitat Jaume I (UJI), E-12071-Castelló de la Plana, Spain; bagut@uji.es (B.A.); pastorm@uji.es (V.P.); 2 Departament de Ciències Agràries i del Medi Natural, Unitat Associada d’Entomologia IVIA-UJI, Universitat Jaume I (UJI), Campus del Riu Sec; E-12071-Castelló de la Plana, Spain

**Keywords:** *Tetranychus urticae*, plant defence, spider mites, herbivore-induced plant volatiles, indirect defences

## Abstract

*Tetranychus urticae* (*T. urticae*) Koch is a cosmopolitan, polyphagous mite which causes economic losses in both agricultural and ornamental plants. Some traits of *T. urticae* hamper its management, including a short life cycle, arrhenotokous parthenogenesis, its haplodiploid sex determination system, and its extraordinary ability to adapt to different hosts and environmental conditions. Currently, the use of chemical and biological control are the major control methods used against this mite. In recent years, some studies have focused on plant defence mechanisms against herbivores. Various families of plant compounds (such as flavonoids, glucosinolates, or acyl sugars) have been shown to behave as acaricides. Plants can be induced upon appropriate stimuli to increase their resistance against spider mites. This knowledge, together with the understanding of mechanisms by which *T. urticae* detoxifies and adapts to pesticides, may complement the control of this pest. Herein, we describe plant volatile compounds (VOCs) with repellent activity, and new findings about defence priming against spider mites, which interfere with the *T. urticae* performance. The use of VOCs and defence priming can be integrated into current management practices and reduce the damage caused by *T. urticae* in the field by implementing new, more sustainable crop management tools.

## 1. Introduction

Spider mites (Acari: Prostigmata: Tetranychidae) are the most important family of plant-feeding mites with agricultural relevance [1,2]. The minute members of this family (200 to 900-µm long) received this name due to their ability to produce silk threads with various functions, including protection and the dispersal from overexploited habitats to new ones [3]. Colonies of several webbing species live within the web, which may become a real nest, e.g., with the persea mite, *Oligonychus perseae* Tuttle, Baker, and Abbatiello [4], discouraging natural enemies from entering and mitigating adverse climatic conditions, including the interception of dangerous UV light [5]. More than 1200 spider mite species have been described, and approximately 10% of them can become agricultural pests [6,7,8]. Among these species, the two-spotted spider mite *Tetranychus urticae* Koch is considered the most serious one [8]. This species is cosmopolitan and polyphagous. Approximately 4000 host plant species have been described worldwide [8], and many of them are important crops, including fruit trees, vegetables, and ornamentals, where *T. urticae* is frequently considered a key pest [9,10]. *T. urticae* regularly feeds on the mesophyll cells on the underside of the leaves, where it is protected from UV light [11]. Therefore, this mite produces mechanical damage consisting of empty cells that results in a dull colour of the affected organ, which may later become blackish as the number of necrotic cells increases. Furthermore, the feeding activity alters cell contents, resulting in lower concentrations of nitrogen, phosphorous, and protein, and disrupts cell physiology, reducing photosynthesis and injecting phytotoxic compounds that decrease yields [12]. The economic impact of *T. urticae* arose after World War II [13], and a plausible explanation of this change is based on the disruption by pesticides of existing natural biological control, mostly exerted by Phytoseiidae predatory mites [14]. However, additional causes cannot be neglected, and in the following pages, we will discuss both intrinsic and extrinsic factors that may have contributed to the increasingly important pest status. Only a thorough knowledge of them will allow us to make the right decisions that can lead to better control of this mite. Particularly, studies focused on *T. urticae*–plant interactions may pave the way for novel approaches to improve existing integrated pest management (IPM) programmes for crops where this mite is a key pest.

The two-spotted spider mite displays characteristics that make it a relevant agricultural pest, such as a short life cycle and adaptive abilities. However, inadequate control methods have lead the prevalence of this phytophagous mite. Strategies developed by spider mites to detoxify acaricides, one of the factors contributing spreading the pest worldwide, have been reviewed previously [2,10]. We will first discuss these intrinsic factors, and then will focus on current management practices, before finally showing how, in accordance with the general philosophy of IPM, natural mortality factors acting on *T. urticae*, through both top–down and bottom–up regulation mechanisms, could be exploited to increase the resilience of our cropping systems. 

## 2. Biological Characteristics of *T. urticae* that Favour Its Development into a Devastating Pest of Crops

### 2.1. Life Cycle and Reproduction

*T. urticae* is an r-strategist [15]. *T. urticae* has most of the traits that characterize r-selected species, such as small size, short life cycle, early sexual maturity, and high offspring production [16]. During their life, *T. urticae* females can produce over 100 eggs at 25 °C, and they measure 490–515 µm long as adults [17]. In optimal conditions, their developmental time is less than a week. Furthermore, *T. urticae* reproduces through arrhenotoky [17], a form of parthenogenesis in which unfertilised eggs develop into males. Males are produced parthenogenetically, while diploid females are usually produced biparentally from fertilised eggs. Reproduction is further altered by the endosymbiont bacterium *Wolbachia* spp. [18]. This endosymbiont has various functions when present in *T. urticae*, from the manipulation of host reproduction to interactions in nutritional and metabolic pathways, interference in development and lifespan, and protection from pathogens and parasites [19,20,21,22,23].

### 2.2. High Ability to Adapt to Harsh Environmental Conditions

*T. urticae* shows different traits that allow it to overcome adverse conditions, such as diapause, which can be defined as a genetically determined state of suppressed development that is controlled by environmental factors [24]. Many changes occur when *T. urticae* enters diapause: it stops feeding and becomes positively geotactic and negatively phototactic in order to find protected sites that can be used as hibernation sites [25,26]. Recently, some authors have described biochemical changes during diapause. A genome-wide microarray used by Bryon et al. [27] revealed changes in pathways implicated in digestion, detoxification, cryoprotection, carotenoid biosynthesis, and the organisation of the cytoskeleton. Using metabolomic approaches, Khodayari et al. [28] found high levels of glucose and gluconolactone in diapausing females. These sugars can be reduced to polyols that contribute to low-temperature tolerance. This knowledge may help refine the control of *T. urticae*, because winter survival in temperate climates has important consequences for pest prevalence [27].

The two-spotted spider mite has a wide range of hosts and can feed on many plant species [8]. To defend against herbivore attacks, plants produce a range of compounds with antixenotic effects, including metabolites with acaricidal activity (α-pinene, sesquiterpenes, and carvacrol, among others). *T. urticae* can detoxify many of these toxic compounds. Dermauw et al. [29] found that host plant adaptation was due to specific protein families with detoxification properties, such as P450 monooxygenases or ring-splitting dioxygenase. When spider mites were moved to a different host plant, the implicated genes were up-regulated. Recently, several publications [30,31,32] report that host plant adaptations affect both the physiology of the mite and the plant defence response. Wybouw et al. [31] showed that plant defence responses were attenuated upon feeding of an adapted strain of *T. urticae* on tomato, whereas non-adapted *T. urticae* induced major changes in defence responses. The expression of cathepsin B and L, legumanins, and aspartyl protease genes seems to play a relevant role in detoxifying toxic compounds in the gut of *T. urticae* [33]. Adult female *T. urticae*, after feeding on Arabidopsis thaliana plants overexpressing a plant protease, increased the expression of those genes. Digestive proteases in *T. urticae* might act as a first defence barrier in the gut against plant defensive proteins [33]. 

In addition to detoxification, dispersal is a key point in the emergence of the pest. *T. urticae* colonisation usually begins with a mated female, followed by rapid population growth, and finalizes when the host plant is overexploited. When the food source becomes scarce, spider mites need to disperse. This species displays some adaptations that enhance its dispersal. When the host plant deteriorates, a wide range of plant hosts increases the probability of finding a suitable new host. During this stage, males, which are smaller and usually less abundant than females, become even scarcer, while the number of female eggs and male mortality increases [6]. Mated females can disperse by crawling [34]. This kind of behaviour normally occurs in parts of the same host plant or in dense aggregations of host plants. However, dispersal from overexploited hosts is usually aerial [35,36]. In this case, spider mites affix a thread to the substrate, hang from the thread in the air, and are carried off by the wind. Likewise, when resources become scarce, individuals can disperse together by the formation of silk balls [37] that can contain thousands of mites. While individual modes of dispersal (crawling and aerial dispersal) are restricted to mated females, silk balls mainly contain immature individuals.

### 2.3. Detoxification and Adaptation to Pesticides

An important trait that makes the control of *T. urticae* difficult is its ability to develop resistance against acaricides in a short time [38,39]. There are several ways by which herbivorous arthropods can become resistant to pesticides, and pests can exhibit more than one resistance mechanism at the same time. Mites can break down pesticides into nontoxic compounds, but they can also avoid the toxin by a change in their normal activity. However, the penetration resistance favours that a resistant population absorbs the toxin slower than susceptible populations, favouring also the selection of resistant populations that in addition may develop alterations in the target of the pesticide [38]. More than 500 cases of resistance have been reported for *T. urticae* [40], including 94 active substances with different modes of action [41].

## 3. Current Management Tools to Control *T. urticae*

In natural ecosystems, spider mite colonies consist of small groups of individuals in equilibrium with their predators, and hardly ever cause significant damage to their host plant [6]. Therefore, the following questions arise: why does *T. urticae* become uncontrolled in agroecosystems, and why does it cause severe economic losses for farmers? To answer these questions, we must consider that there is no single reason, but rather several that contribute to the increase in the potential damage done by *T. urticae*. According to Rabbinge [42], the crops suffering most from spider mite damage in 1985 were fruit orchards, as well as many ornamental and horticultural crops both under glasshouse and in open field conditions. Currently, among mite pests, the most economically relevant losses can still be attributed to *T. urticae* [10].

Management of *T. urticae* in these systems has for a long time been primarily based on chemical control [43,44]. Surprisingly, severe widespread outbreaks of *T. urticae* populations occurred during the 1950s [43]. This occurrence coincided with the generalisation of pesticide control as the primary means to suppress pest populations in increasingly productive crops, which became high-quality food sources for spider mites. Most of these wide-range long-persistence pesticides are currently either banned or are not recommended under IPM labels in developed countries, mainly due to toxicological and environmental issues. For instance, in the European Union (EU), of the 103 active substances included in the EU database as acaricides that were once authorised in the EU member states, only 32 are presently approved [41]. Specific, more selective modes of action that centre on the inhibition of basic mite functions such as growth, mitochondrial respiration, or lipid biosynthesis have been gaining importance [45]. The sustainable use of these acaricides in combination with additional control methods can be achieved by treatments based on the use of sampling plans and economic injury levels when possible [46], and the rotation of compounds with different modes of action groups in order to delay the selection of resistance to any one type of acaricide as much as possible [41].

Biological control methods mostly depend on the use of entomopathogens and entomophagous arthropods. Although species may vary locally, taxonomic groups often coincide worldwide, even at the genus level [47]. The most effective group of *T. urticae* natural enemies, as well as for all tetranychids, belongs to the Phytoseiidae mite family. Indeed, some IPM programmes include sampling and manipulation of their populations through augmentative release and conservation [48]. Additionally, less frequent predators can be found in the mite families Anystidae, Bdellidae, Cheyletidae, Erythraeidae, and Stigmaeidae [49]. There are more than 2000 species of phytoseiids in 67 different genera [49]. Some Phytoseiidae are almost specialists on *T. urticae* (e.g., *Phytoseiulus persimilis* (Athias-Henriot)), whereas others can feed on other Tetranychidae as well (e.g., *Neoseiulus californicus*), and some phytoseiids are generalist predators that may include *T. urticae* in their diet (e.g., *Euseius* spp.) [50]. In addition to mites, some insect predators from different orders can also effectively feed on *T. urticae*. The most important ones, which in some cases are commercially available, include the Coccinellidae *Stethorus punctillum* Weise, the Neuropterans *Chrysoperla* spp. (Chrysopidae), *Conwentzia* spp. and *Semidalis* spp. (Coniopterygidae), predatory thrips such as *Scolothrips longicornis* Priesner (Thysanoptera: Thripidae) and the midge *Feltiella acarisuga* (Vallot) (Diptera: Cecidomyiidae).

### 3.1. Secondary Pest Outbreaks Triggered by Unsuitable Management

Secondary pest outbreaks can be caused by broad-spectrum insecticides that disrupt natural pest control in different ways. Direct effects due to the toxicity of the insecticide on non-target natural enemies (in this case, mostly Phytoseiidae mites) are expected [13]. However, insecticides can also cause indirect impacts on the natural enemies as their food resource is reduced or the prey is contaminated [51,52]. Moreover, many insecticides have a higher negative impact on natural enemies than the pestiferous species on which these natural enemies feed [53,54,55,56,57,58]. The overuse of insecticides in crops can transform a harmless arthropod into a serious economic problem. In fact, the mite pest *Panonychus ulmi* (Koch) became a serious problem when its biological control by *Amblyseius fallacis* (Ga rman) was interfered with by pyrethroids [59,60].

An additional factor that may prevent the correct control of mites in agricultural ecosystems is the stimulating effect of some insecticides on diverse biological parameters, the so-called hormoligosis phenomenon [61]. For instance, James and Pryce [62] showed that sub-lethal doses of imidacloprid caused a 20% increase in the number of eggs produced by *T. urticae* compared with water treatment. Likewise, Marcic [63] measured the intrinsic rate of increase of *T. urticae* growth after sub-lethal doses of clofentezine, and observed a significant increase compared to water-treated females.

Finally, the emergence of spider mites as a major pest is related to the plant domestication process, where crops have been selected based on traits such as yield or palatability at the expense of pest-resistance traits [64,65]. In most cases, breeding programmes to improve crops are focussed on reaching acceptable commercial aesthetic standards that are rarely linked to resistance to pests and diseases [65]. Therefore, the cultivars selected usually display increased susceptibility to pests. 

## 4. Plant Defence Mechanisms that Contribute to a Sustainable Control of *T. urticae*

The development of new acaricides with no side effects on non-target organisms, including natural enemies, can be complemented with an adequate handling of plant innate immunity. However, the knowledge of plant responses to *T. urticae* is still limited, and further research is needed.

Upon arthropod herbivore attack, plants present a battery of defences that either reduce pest abundance or increase plant tolerance to injury. Three major defence mechanisms are recognised to contribute to resistance against arthropods: antibiosis (e.g., reduced fecundity, longevity, or survival); antixenosis, which affects the behaviour of the pest, and tolerance, which refers to plant recovery following an attack [66,67].

Plant basal resistance against arthropods is gradual, and results from a combination of these three defence mechanisms. Plants have evolved different effective mechanisms, such as physical barriers or chemical defences that can be constitutive or inducible. Direct defences such as thorns, prickles, or high levels of lignification directly promote a detrimental effect on the herbivore. Another cue for direct defences are the secondary metabolites, which can be toxic to the arthropod, e.g., by interfering with digestive processes. The potential of plant defence mechanisms against *T. urticae* can be exploited in IPM programs.

### 4.1. Pre-Existing Constitutive Defences against T. urticae

Constitutive defences include all those barriers that are present in the plant before the challenge appears. They constitute the first shield that the herbivore arthropod confronts [68]. 

Glandular and non-glandular trichomes are one of the first and powerful defences present. They can accumulate secondary metabolites in addition to having a role in physically blocking the establishment of the predator. Glandular trichomes play a role as chemical defences because they can secrete toxic compounds [69,70], whereas the function of non-glandular trichomes is to impair the establishment and movement of small arthropods on the leaf surface, and hinder the access to the epidermis for feeding [71]. In the case of the raspberries, *Rubus idaeus*, leaf trichomes have a deterrent effect on *T. urticae* [72].

Plant cuticles can also act as physical barriers. They are formed by polymeric lipids and soluble waxes that cover the leaf tissues. Plants with glossy surfaces and reduced wax blooms are usually resistant to smaller arthropods [73].

As chemical constitutive defences, plant secondary metabolites and toxic proteins play an important role in defence against pests. For example, some chemicals can be stored in cell compartments before a biotic stress and can be released, once the arthropod wounds the leaf and the cell membranes are disrupted. These chemicals are known as phytoanticipins [74]. This is the case with mustard oil [75,76]. In this regard, non-adapted strains of *T. urticae* have been shown to be sensitive to the toxicity of Arabidopsis glucosinolates [77]. These studies will be discussed in Section 4.2.2.

### 4.2. Inducible Defences against T. urticae

#### 4.2.1. Hormone Signalling Pathways Activated Upon Mite Infestation

Plants can activate diverse pathways controlled by phytohormones, including abscisic acid (ABA), salicylic acid (SA), oxylipins (jasmonic acid and JA derivatives), and ethylene (ET) in response to biotic attack. Plant hormones regulate signalling pathways to produce secondary metabolites and defensive proteins that have a detrimental effect on various biological parameters of the herbivore. For an appropriate hormonal response regarding timing and quantity, recognition of the attack by a spider mite is extremely relevant. However, knowledge about these first stages of spider mite infestation is limited. In tomato, the polyphagous *T. urticae* Koch DeLier-1 and the Solanaceae-specialist *Tetranychus evansi* Baker and Pritchard can suppress SA and JA-dependent responses, although these mechanisms seem to be time dependent [78]. In fact, the mite *T. evansi* can suppress JA-dependent responses by stimulating the SA pathway, activating negative cross-talk between these hormones [79,80]. Non-adapted strains of *T. urticae* induce both JA and SA-dependent defences, whereas the specialist *T. evansi* suppresses a larger subset of genes activated by *T. urticae* [78].

It was recently discovered that salivary secreted proteins can suppress responses downstream of SA and JA pathways. The transient expression of three proteins discovered in the secretome of *T. urticae* in *Nicotiana benthamiana* improved the performance of the mite. Despite the fact that some proteins, such as TE8, may act as elicitors to recognize an attack, other proteins such as TU28, TU84, and TC84 may function as effector proteins, suppressing plant response [81]. The feeding mechanism of *T. urticae* has been recently described, and this may also explain how the plant is manipulated by this mite. The stylet of *T. urticae* usually penetrates through stomata or epidermal pavement cells to reach single mesophyll cells [82]. Mites avoid epidermal damage, and this may contribute to minimizing detection of the attack by the leaf surface, therefore delaying the plant response. This is clear evidence that the Pathogen-Associated Molecular Pattern (PAMP)-triggered immunity (PTI) and the effector-triggered susceptibility (ETI) described for pathogens is also functional in plant–arthropod interactions [83]. In this case, Mithöfer et al. described the herbivore-triggered immunity (HTI) that is initiated by herbivore-associated molecular patterns (HAMPs) [84]. Interestingly, the damage caused by herbivore attack leads to a change in the plasma membrane potential and subsequent cytosolic free Ca^2+^ changes, triggering a signal that activates a cascade of events. Arimura et al. [85] demonstrated that a chelator of extracellular Ca^2+^ blocks the defence responses of lima bean plants after spider mite infestation. Another rapid event after attack is the production of reactive oxygen species (ROS). However, the role of H_2_O_2_ in the signal transduction against spider mites remains unclear, because Leitner et al. [86] reported the production of H_2_O_2_ only at late stages of arthropod attack.

Following ROS production, a major regulatory signal during herbivore attack is the hormone JA. Various studies have reported activation of the JA pathway after mite infestation in tomato [87,88], Arabidopsis [89], and citrus [90,91]. In these plant species, the JA activation occurs only one or two days after infestation, whereas in *Medicago truncatula*, the increase in JA levels occurs only in yellowing leaves [86]. These differences may be a consequence of the plant’s recognition of the spider mite attack, and Arabidopsis and citrus respond faster by activating JA-dependent signalling. In addition to JA, SA-mediated signalling is also activated in some plant–herbivore interactions [87,88,91]. Although both hormones can be induced following mite attack, they play different roles in the plant–mite interactions. Agut et al. [91] showed that methyl jasmonate (MeJA) application to susceptible citrus plants could re-establish resistance in a highly susceptible phenotype, whereas SA application did not promote resistance. Zhurov et al. [89] also showed the relevance of the oxylipin pathway as a major defence against mites. Accordingly, Arabidopsis *aos* (impaired in JA biosynthesis) and *myc2–4* (impaired in JA response) mutants show increased susceptibility to spider mite attack due to faster development from larvae to nymph and reduced larval mortality.

**Figure 1:**
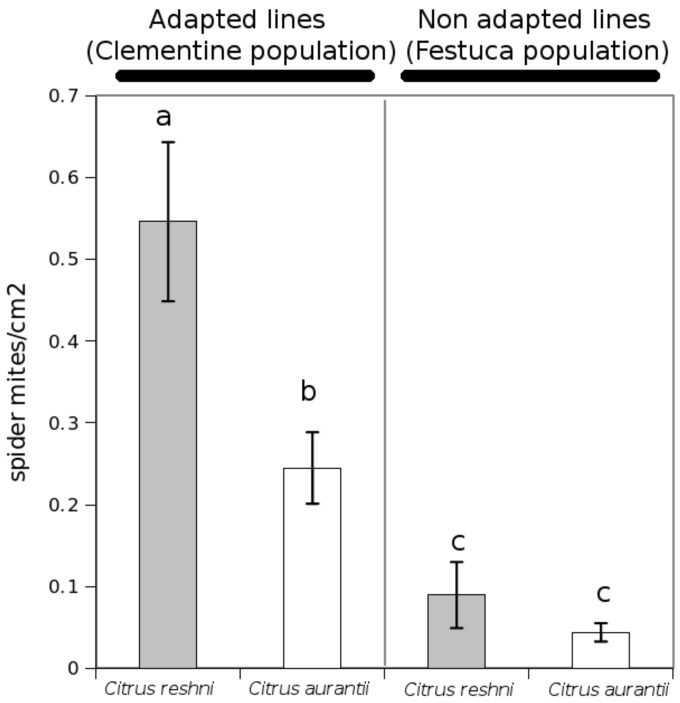
Plant resistance to adapted and non-adapted lines of *T. urticae*. Two different citrus genotypes, *C. reshni* and *C. aurantium*, are susceptible and resistant, respectively, against *T. urticae* when this mite was originally reared on citrus (Clementine) leaves for more than 30 generations. However, both citrus genotypes are strongly resistant to the infestation of mite lines grown in *F. arundinacea* leaves for more than 30 generations. Three-month-old plants were infested with five adult females mites per plant, and the number of adults was scored 14 days after infestation.

*T. urticae* breeding for up to 30 generations on tomato leaves significantly increased its growth rate and performance [31]. This increase is explained by two major factors: a) adaptation of the mite by enhancing the expression of genes that encode detoxifying enzymes and xenobiotic transporters, and b) adapted mite effectors that interfere with the plant response, suppressing defences, compared to non-adapted strains of the mite. This strongly suggests an increased ability of the mite to hide its presence from the host. Host manipulation by mite effectors has been suggested to occur in the case of *T. urticae* [78,82,92], and a clear example can be observed in the experiment shown in Figure 1. The citrus genotype *Citrus reshni* is susceptible compared to *Citrus aurantium* when plants are infested with a citrus-adapted strain of *T. urticae*. Surprisingly, both genotypes are strongly resistant when a *Festuca arundinacea*-adapted strain was used to infest plants (Figure 1). Whether successful mite adaptation to the host depends on phytoalexin detoxification or on host manipulation is still a matter of debate [77]. Although effector proteins from *T. urticae* are still under study, a very recent discovery demonstrates the enormous plasticity of the mite to modify the protein content of its saliva, which changes the composition depending on the host plant [93].

Although it is almost impossible to find a single herbivore attack in real field conditions, little is known about plant responses to multiple herbivore attacks. Glas et al. [94] demonstrated that *T. urticae* colonizes plants already infested with *Aculops lycopersici* (Massee) (Acari: Eriophyidae) with greater intensity. A. lycopersici induced SA responses in tomato plants that suppress the JA pathway. In contrast, there are also examples where *T. urticae* did not benefit from an interspecific infestation. For example, the mirid bug *Macrolophus pygmaeus* (Rambur) is a zoophytophagous biological control agent that is used against whiteflies, aphids, and spider mites [95]. *M. pygmaeus* also feeds on plants, triggering increases in the levels of proteinase inhibitors in local and systemic tissue, which negatively impact the *T. urticae* performance [96].

#### 4.2.2. Plant Secondary Metabolites in Plant Defence against *T. urticae*

Even though plant secondary metabolites in many cases are under the control of phytohormones, they deserve attention, because they have been described as highly effective in the defence against arthropods.

Among the secondary metabolites that play a relevant role in defence, terpenes and terpenoids are under the regulation of the JA pathway along the plant–pathogen–insect interaction axis [97,98]. They mediate plant antixenosis, as well as plant antibiosis. Terpenes are commonly found in essential oils at high concentrations; *Lippia sidoides* Cham. (Verbenaceae) oils produce a negative effect on *T. urticae* [99]. Among the terpenoids found in essential oils, thymol and carvacrol show potent acaricidal activity. In tomato, the terpenoid sesquiterpene 7-epizingiberene reduces mite fecundity, and therefore affects population densities [100].

Another group of chemicals with insecticidal properties are the aromatic and aliphatic glucosinolates that are commonly found in Brassicaceae. These compounds are highly concentrated in mustard oil, and are highly toxic to arthropods. These oils contain sugar glucosinolates that are cleaved by myrosinases when the plant leaf is chewed, cut, or otherwise damaged [101,102,103,104]. Zhurov et al. [89] reported a synergistic interaction between JA and glucosinolates in the Arabidopsis–*T. urticae* interaction. Non-adapted lines of mites performed better on JA-impaired Arabidopsis mutants. However, mite attack also increased the expression of genes related to tryptophan catabolism and indoleacetic acid biosynthesis, which gives rise to indolic glucosinolates (IG). Using metabolomic approaches, it was demonstrated that some IGs such as I3M (indol-3-ylmethyl glucosinolate), 1-MeO-I3M (1-methoxy-indol-3-ylmethyl glucosinolates, and neoglucobrassicins), and 4-OH-I3M (4-hydroxyindol-3-ylmethyl glucosinolate) levels were higher following spider mite infestation. Accordingly, the relevance of IGs in defence was assessed using IG mutant lines on which the development of *T. urticae* was increased. A microarray analysis of spider mites reared on IG mutants or wild-type plants showed that the increased expression of detoxification genes such as P450 monooxygenases, glycosyltransferases, and lipocalins correlated with IG levels [89,105]. Note that the implication of glucosinolates was demonstrated using lines grown on *Phaseolus vulgaris*. According to the innate ability of *T. urticae* to detoxify xenobiotics and its fast host adaptation ability, these experiments would have benefitted from using mites reared on Arabidopsis [89].

Both terpenes and glucosinolates have been shown to be related to tomato and Arabidopsis resistance to *T. urticae*. Interestingly, the analysis of different plant–*T. urticae* interactions, such as with citrus, has shed light onto the new secondary metabolites that play a relevant role in plant defences against this mite. Among these, flavonoids were found to be highly overaccumulated in resistant citrus genotypes following mite infestation [91]. Metabolomic analysis revealed higher levels of flavonoids such as naringenin, hesperetin, and p-Coumaric acid (a precursor of flavonoids) in resistant versus susceptible genotypes. Additional experiments showed that treatments with a blend of all three compounds can effectively protect citrus plants against the mite, whereas individual treatments did not reduce mite oviposition [91]. These observations suggest that, at least, citrus defence is based on a complex multicomponent defence. The expression of chalcone synthase (*CHS*), a key gene in the synthesis of flavonoids, was also enhanced in the resistant genotype following infestation by the mite. The regulation of flavonoid biosynthesis has also been proposed to be under JA pathway control [106,107]. Interestingly, the alkaloid macarpine, which is derived from shikimate, was found in both resistant and susceptible genotypes upon priming treatment [108]. Priming is a physiological phenomenon of plant immune adaptation by which a plant can react faster and stronger to a biotic or an abiotic stressor [109,110].

Acyl sugars are another set of defence secondary metabolites. These compounds are not intracellular, but are rather produced and secreted from glandular trichomes on the plant leaf and stem surface, providing a sticky feel to plant tissues. Acyl sugars provide physical and/or chemical defence to many plant species in the Solanaceae family [111]. The density of type IV glandular trichomes and the production of acyl sucrose are correlated with increased mortality, repellence, and reduced oviposition in *T. urticae*. Notably, acyl sugars seem to also be controlled by the JA pathway [112]. Accordingly, MeJA applications in *Datura wrightii* (Solanaceae) increased the production of these compounds by 44%.

#### 4.2.3. Defensive Proteins and Peptides against T. urticae

In addition to secondary metabolites, plants can also synthesise defensive proteins with various structures and functions. Proteinase inhibitors (PIs), a family of digestive proteases that act in the insect midgut, have been widely studied as components of plant defence [113,114]. They act in the herbivore gut, inactivating proteases and disrupting digestive processes, inducing amino acid deficiencies that negatively affect the performance of the herbivore [115,116,117]. Carrillo et al. [118] characterised a barley cystatin gene that codifies a phytocystatin (inhibitor of cysteine protease). Transgenic maize plants overexpressing the barley cystatin *LCY6* gene showed reduced reproduction and increased development time of *T. urticae*. Seemingly, Arabidopsis plants carrying both the cystatin Icy6 and the trypsin inhibitor Itr1 showed reduced leaf damage compared with wild-type plants [119]. *T. urticae* feeding on these transformant lines presented less cathepsin B activity, which participates in protein catabolism. A reduction in the cathepsin activity could promote amino acid deficiencies with detrimental effects on the performance of spider mites. Recently, a MATI (mite attack triggered immunity) protein has been described as a strong regulator of Arabidopsis resistance against spider mites [105]. The gene was identified because it accumulates in the Arabidopsis resistant accession Bla-2 relative to the susceptible Kon accession. This protein modulates sulphur and photosynthetic pigments, and thus redox homeostasis, as well as phytohormone signalling pathways upon *T. urticae* infestation. In fact, this mite causes more leaf damage on a MATI knockdown mutant than in the Col–MATI overexpressing line. Moreover, the relevance of this protein in plant protection and defence is also supported by the same phenotypic response after an attack by a chewing insect.

#### 4.2.4. Induced Responses Triggering Direct Systemic Defence against T. urticae

There is a growing interest in understanding induced resistance against herbivores [120]. In this regard, systemic signals that induce resistance are under active research. Previous research has shown that an interaction with below-ground herbivores can promote enhanced defence against above-ground herbivores and fungi [121]. Karban and Carey [122] showed that cotton plants that experienced an infestation by *T. urticae* had reduced mite populations, which is an example of herbivore-induced resistance (HIR) triggered by mites. Unfortunately, these experiments did not provide molecular evidence to explain these observations. Although it is still a matter of discussion, distal signalling in response to herbivores and wounding seems to be transmitted through an electrical signal. The damage produced by the herbivore induces membrane depolarisation, and this electric signal is transmitted to systemic undamaged leaves. The electrical signal is perceived at the distal tissue by the glutamate receptor-like GLR protein. Following recognition, activation of the JA pathway occurs [123]. Recent studies using grafting experiments indicate this is a very fine-tuned response in citrus exposed to *T. urticae* [124]. An unknown wounding signal produced in the shoot is transmitted to the roots, which respond accordingly, releasing a pool of mobile secondary metabolites. Among them is glutamic acid, which triggers the priming of *LOX2* gene expression and activates GLR in the shoots. Furthermore, these signals, as well as the resistance, are graft-transmissible when the root system belongs to a resistant genotype. 

#### 4.2.5. Herbivore-Induced Plant Volatiles (HIPVs) in Plant Resistance

The release of antixenotic herbivore-induced plant volatiles (HIPVs) by mite-attacked plants may have two interpretations. On the one hand, it may be understood as a defensive response to avoid further attacks. On the other hand, the mites may use this blend to understand that a given host is a poor source of nutrients. Particularly in the case of *T. urticae*, the data are still controversial. Dicke [125] showed that HIPVs emitted by lima bean (*Phaseolus lunatus*) plants are a repellent for *T. urticae*, whereas Pallini et al. [126] showed that mite-infested cucumber (*Cucumis sativus*) plants were more attracted to conspecifics. In addition to the different relative basal resistance of both hosts, given the extremely efficient adaptation of *T. urticae* to diverse hosts, these investigations must be explored carefully, because experimental conditions or host adaptation may explain such apparent differences in the role of HIPVs. Furthermore, the level of infestation is also an important factor, because the mites may detect an overexploited host. In these cases, they may prefer clean, uninfested plants. Agut et al. [108] showed that uninfested resistant and susceptible citrus genotypes are equally attractive for *T. urticae*. Interestingly, the choice of the mite when the plant has experienced a previous infestation is totally different, as they prefer the susceptible genotype. These observations suggest either innate or learning behaviour of the mite that identifies odours that make a host more attractive or repellent. Despite these observations, the specific role of volatiles implicated in attraction/repellence needs to be further studied.

The volatiles released by infested plants can warn distal plant parts, as well as neighbouring plants, thereby priming defences. In plant–pathogen interactions, the priming phenomenon has been extensively studied, but in plant–herbivore interactions, knowledge in this field is still limited [127]. In 1983, Baldwin and Schultz [128] showed the first evidence that HIPVs could be involved in defence activation or priming in nearby plants. Maize plants after (*Z*)-3-hexen-1-ol (GLV) exposure showed primed levels of JA [129] and a more efficient defence response. A similar response was described in lima bean plants after the exposure of volatiles from tobacco transgenic lines [130]. Plants that overproduce β-ocimene are more resistant to *T. urticae*, and in turn are more attractive to predatory mites. Therefore, the role of volatiles in plant resistance against mites, and particularly in priming, is not restricted to direct defences but rather also involves indirect defences by attracting beneficial predatory mites. The GLV α-ocimene, originating from infested *Citrus aurantium* plants, has also been shown to induce resistance to *T. urticae* in the susceptible genotype *C. reshni* [108], leading to reduced oviposition. This volatile already overaccumulates at basal levels (without infestation) in *C. aurantium* plants, which is the resistant rootstock, and may be a good candidate to induce resistance against *T. urticae*, since conspecific mites avoid infested *C. aurantium* plants. In addition to this volatile, others such as α-farnesene, d-limonene, 4-hydroxy-4-methyl-2-pentanone, benzoic acid 2-(methylamino)-methyl ester, and MeJA accumulate in infested *C. aurantium* plants. Agut et al. [109] found that some HIPVs released by *C. aurantium* plants induce *LOX2* gene expression in *C. reshni*, although this expression was concentration dependent. *C. reshni* exposed to *C. aurantium*-HIPVs induced the SA marker PR5 and the flavonoid synthesis gene *CHS*, whereas the oxylipin pathway marker PR3 was highly down-regulated, suggesting complex defence regulation depending on the genotype. Additionally, the metabolite macarpine also overaccumulates in the susceptible *C. reshni* that is exposed to *C. aurantium* HIPVs.

## 5. Top–Down and Indirect Defence Mechanisms against *T. urticae*

The top–down and indirect defence mechanisms involve a third trophic level (the natural enemies) that interacts with the plant and the pest [131]. Several control techniques based on these tritrophic interactions have been developed. Two major methods are employed to increase the number of natural enemies in the field: first, the release of attractants for natural enemies, and second, the improvement of food and shelter provisioning. This is known as the conservation of biological control, because both methods provide a safe environment for predator preservation [68,132,133].

Predatory mites are known to be attracted by HIPVs such as methyl salicylate (MeSA) [134]. In fact, a specialist predator of Tetranychus spp., *Phytoseiulus persimilis*, is equally attracted to MeSA and the plants that are infested with its prey [135]. The absence of MeSA interferes with *P. persimilis* choice, and it can be restored by complementary treatments of MeSA. Another important predatory mite of the two-spotted spider mite is *Neoseiulus californicus*, a specialised predator of tetranychid mites. In lima bean plants infested with *T. urticae* MeSA, linalool and three green-leaf volatiles (GLVs), (*Z*)-3-hexen-1-ol, (*Z*)-3- hexenyl acetate, and (*E*)-2-hexenal have been identified. This blend is highly attractive to *N. californicus*. However, when MeSA is removed from the blend, the attraction is lost.

In other plant species, the role of the MeSA as an attractant of predatory mites remains unclear. Kappers et al. [136] showed that differences in volatile production by different cultivars of cucumber had an impact on the preference of P. persimilis. The levels of (*E*)-β-ocimene and (*E*,*E*)-TMTT (homoterpene (*E*,*E*)-4,8,12-trimethyltrideca-1,3,7,11-tetraene) were correlated positively with the attraction of predatory mites. In contrast, a negative correlation was found between MeSA and predator–mite attraction. Thus, it is likely that the other components of the blend also modulate the final output in the phytoseiid behaviour.

In plants with multiple infestations, the role of terpenes is more complex. β-ocimene is released by lima bean plants that have been infested with *T. urticae*. However, a subsequent infestation with the whitefly *Bemisia tabaci* (Gennadius; Homoptera: Aleyrodidae) interferes with the recognition by *P. persimilis* [97], but when β-ocimene was added to the plants with *T. urticae* and *B. tabaci*, the attraction of the predatory mite was restored. These results are the consequence of the activation of multiple pathways. The attack by *B. tabaci* increases the levels of SA in lima beans. The SA increase blocks the JA pathway, suppressing the β-ocimene synthase that is regulated by JA levels, and therefore the production of β-ocimene is reduced.

Another terpenoid with a role in tritrophic interactions is the homoterpene (*E*,*E*)-TMTT. *Lotus japonica* overproducing (*E*,*E*)-TMTT displays an increased attractiveness to *N. californicus* [137]. In contrast, *P. persimilis* did not show a preference in a choice test where wild-type or (*E*,*E*)-TMTT plants were offered. This difference may be due to the feeding habits of these predators. *P. persimilis* is a voracious and specialised predator of *T. urticae*, and likely needs a severe infestation to survive. *N. californicus* is a generalist predator that can feed on pollen and other tetranychid mites. For this reason, this mite may also be attracted by uninfested transgenic lines.

The association of plants with arbuscular mycorrhizal fungi (AMF) though mutualistic symbiosis can strongly alter plant physiology [138]. Indeed, this symbiosis can also alter the interaction with mite pests [139,140]. The released terpenoids β-ocimene, DMNT, TMTT, and linalool are elevated in lima bean plants colonised by *Funneliformis mosseae* compared with non-mycorrhizal plants. All of these compounds seem to be responsible for increased preference by *P. persimilis* [141].

Field assays with different synthetic volatile dispensers have been performed to determine the real effect of these volatiles as attractants of natural enemies [142,143,144,145]. Rodriguez-Saona et al. [146] reviewed several studies related to MeSA in field assays. Forty-one out of 91 observations showed a significant attraction. There is evidence that natural enemies are broadly attracted to MeSA in the field; however, further investigations are needed to apply this knowledge to field treatments.

To improve the biological control of *T. urticae*, van Wijk et al. [134] offered predatory mites single volatile compounds from a blend of attractive plants obtained in a field trial. Surprisingly, most of the compounds tested had a result that was the opposite of what was expected. These experiments suggest that predatory mites do not respond to single compounds, but rather to a mixture of volatiles. Thus, the administration of complex blends though dispensers or new programmes of plant breeding for increased volatile production may prove to be good approaches for enhanced biological control in the future.

As already commented in this section, food and shelter provisioning (nectar, pollen, or alternative prey) may also be used to maintain or increase populations of beneficial arthropods [147]. In fact, food provision when the main food resource (the pest) is scarce may help to maintain constant densities of natural enemies. Ground covers can be used to increase food provision to natural enemies, while also providing refuge and hibernation or aestivating sites, which are required in order for predatory and parasitic arthropods to successfully develop to adulthood and reproduce. *Festuca arundinacea* Schreb. (Poaceae) was described by Aguilar-Fenollosa et al. [133] as an effective cover crop that increased the densities of *P. persimilis* and *N. californicus* in citrus. A specific strain of *T. urticae* and a Poaceae-specific thrips species that could be used by these two phytoseiids as alternative prey are commonly found in this cover crop [131,148]. Another relevant parameter to improve the biological control is the provisioning of pollen. However, not all pollen shows the same effectiveness. Pina et al. [149] showed in semi-field experiments that the pollen from *Carpobrotus edulis* (L.) L. Bolus increased the proportion of *Euseius stipulatus*, and this effect impaired the levels of phytoseiid species suffering intraguild predation by *E. stipulatus*, whereas this did not occur in *F. arundinacea* pollen [150,151].

## 6. Conclusions

**Figure 2:**
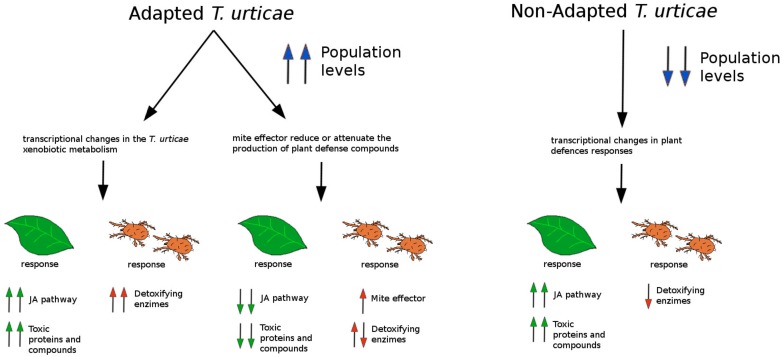
Interplay between host-adapted and non-adapted mites and susceptible and resistant plants. Different lines of *T. urticae* can be found in the field. Some lines can modify the defence mechanisms of the plant by injecting effectors into the host. Mite effectors can interfere in the activation of defence pathways. When the effector is absent from the mite, the plant responds to the infestation by activating effective defences such as the JA pathway. This JA activation gives rise the production and accumulation of terpenoids, glucosinolates, flavonoids, acyl sugars, and other defensive compounds with detrimental effects on the mite. Additionally, green-leaf volatiles (GLVs) and terpenoids can also contribute to resistance and to volatile compounds (VOCs)-IR in distal plant tissues or neighbouring plants. From the mite side, adapted lines of *T. urticae* are extremely efficient detoxifying host chemicals, and may manipulate host responses. This would remove the need for the detoxification of phytoalexins from the host. In contrast, non-adapted strains are recognised by the host, which responds massively by activating defensive compounds and proteins. Finally, the host itself can also be conditioned by defence priming: upon appropriate stimuli, the host can prepare to react faster and stronger to the mite attack, even upon attack by adapted lines of *T. urticae*, and therefore be resistant. Green arrows indicate plant responses, red arrows indicate mite responses, and blue arrows indicate the behaviour of mite populations.

*T. urticae*, a cosmopolitan pest, is a serious problem in many crops and ornamental plants due to a high reproduction rate, dispersion, and the ability to detoxify toxic compounds. In addition to these intrinsic factors, there are some crop management procedures that complicate *T. urticae* control, such as pesticide overuse. The various side effects caused by the use of biocides have made the inclusion of alternative, more sustainable methods (which are basically cultural and biological) a prerequisite of any sound modern approach to managing this mite. Within the context of IPM, which is considered the key approach to modern crop protection [17], effective chemical control methods should be not only effective against the target pest, but also compatible with biological control agents, and safe for other non-target organisms (including humans) and the environment. Figure 2 shows a summary of different plant–mite interactions that are relative to the level of adaptation of the mite and the plant’s defence response. Induced resistance against spider mites is a new and interesting approach that can be harmoniously combined with other existing IPM tools. In fact, the use of priming stimuli to enhance plant defences against arthropods has already been proposed [109,110,127]. Thus, in the near future, the combination of traditional IPM methods with defence priming may contribute to increasing the efficacy of mite control in crop plants. To achieve this goal, a thorough understanding of the defense mechanisms orchestrated by plants at the molecular level is necessary. This will most likely pave the way for more sustainable crop protection practices.

## References

[1] Jeppson L.R., Keifer H.H., Baker E.W. (1975). Mites Injurious to Economic Plants.

[2] Van Leeuwen T., Vontas J., Tsagkarakou A., Dermauw W., Tirry L. (2010). Acaricide resistance mechanisms in the two-spotted spider mite *Tetranychus urticae* and other important Acari: A review. Insect Biochem. Mol. Biol..

[3] Fleschner C.A., Badgley M.E., Ricker D.W., Hall J.C. (1956). Air drift of spider mites. J. Econ. Entomol..

[4] Saito Y., Helle W., Sabelis M.W. (1985). Life types of spider mites. Spider Mites. Their biology, Natural Enemies and Control.

[5] Saito Y. (2010). Plant Mite and Sociality: Diversity and Evolution.

[6] Helle W., Sabelis M.W. (1985). Spider Mites. Their biology, Natural Enemies and Control.

[7] Bolland H.R., Gutierrez J., Flechtmann C.H. (1998). World Catalogue of the Spider Mite Family (Acari: Tetranychidae).

[8] Migeon A., Nouguier E., Dorkeld F. (2010). Spider Mites Web: A Comprehensive Database for the Tetranychidae. Trends in Acarology.

[9] Grbić M., van Leeuwen T., Clark R.M., Rombauts S., Rouzé P., Grbić V., Verdon L. (2011). The genome of *Tetranychus urticae* reveals herbivorous pest adaptations. Nature.

[10] Attia S., Grissa K.L., Lognay G., Bitume E., Hance T., Mailleux A.C. (2013). A review of the major biological approaches to control the worldwide pest *Tetranychus urticae* (Acari: Tetranychidae) with special reference to natural pesticides. J. Pest Sci..

[11] Ohtsuka K., Osakabe M. (2009). Deleterious effects of UV-B radiation on herbivorous spider mites: They can avoid it by remaining on lower leaf surfaces. Environ. Entomol..

[12] Johnson W.T., Lyon H.H. (1991). Insects that Feed on Trees and Shrubs.

[13] Hoy M.A. (2011). Agricultural Acarology: Introduction to Integrated Mite Management.

[14] Huffaker C.B., Vrie M.V., McMurtry J.A. (1969). The ecology of tetranychid mites and their natural control. Annu. Rev. Entomol..

[15] MacArthur R.H., Wilson E.O. (1967). The Theory of Island Biogeography.

[16] Grbic M., Khila A., Lee K.-Z., Bjelica A., Grbic V., Whistlecraft J., Verdon L., Navajas M., Nagy L. (2007). Mity model: *Tetranychus urticae*, a candidate for chelicerate model organism. Bioassays.

[17] Helle W., Gutierrez J., Bolland H.R. (1970). A study on sex-determination and karyotypic evolution in Tetranychidae. Genetica.

[18] Werren J.H. (1997). Biology of wolbachia. Annu. Rev. Entomol..

[19] Moran N.A., McCutcheon J.P., Nakabachi A. (2008). Genomics and evolution of heritable bacterial symbionts. Ann. Rev. Genet..

[20] Brownlie J.C., Johnson K.N. (2009). Symbiont-mediated protection in insect hosts. Trends Microbiol..

[21] Gross R., Vavre F., Heddi A., Hurst G.D., Zchori-Fein E., Bourtzis K. (2009). Immunity and symbiosis. Mol. Microbiol..

[22] Cook P.E., McGraw E.A. (2010). *Wolbachia pipientis*: An expanding bag of tricks to explore for disease control. Trends Parasitol..

[23] Breeuwer J.A., Werren J.H. (1993). Cytoplasmic incompatibility and bacterial density in *Nasonia vitripennis*. Genetics.

[24] Hunter M.D., McNeil J.N. (1997). Host-plant quality influences diapause and voltinism in a polyphagous insect herbivore. Ecology.

[25] Veerman A., Helle W., Sabelis M.W. (1985). Diapause. Spider Mites. Their biology, Natural Enemies and Control.

[26] Foott W.H. (1965). Geotactic response of the two-spotted spider mite, *Tetranychus urticae* Koch (Acarina, Tetranychidae). Proc. Entomol. Soc. Ont..

[27] Bryon A., Wybouw N., Dermauw W., Tirry L., van Leeuwen T. (2013). Genome wide gene-expression analysis of facultative reproductive diapause in the two-spotted spider mite *Tetranychus urticae*. BMC Genomics.

[28] Khodayari S., Moharramipour S., Larvor V., Hidalgo K., Renault D. (2013). Deciphering the metabolic changes associated with diapause syndrome and cold acclimation in the two-spotted spider mite *Tetranychus urticae*. PLoS ONE.

[29] Dermauw W., Wybouw N., Rombauts S., Menten B., Vontas J., Grbic M., Clark R.M., Feyereisen R., Van L.T. (2013). A link between host plant adaptation and pesticide resistance in the polyphagous spider mite *Tetranychus urticae*. Proc. Natl. Acad. Sci. USA.

[30] Martel C., Zhurov V., Navarro M., Martinez M., Cazaux M., Auger P., Migeon A., Santamaria M.E., Wybouw N., Diaz I. (2015). Tomato whole genome transcriptional response to *Tetranychus urticae* identifies divergence of spider mite-induced responses between tomato and Arabidopsis. Mol. Plant Microbe Interact..

[31] Wybouw N., Zhurov V., Martel C., Bruinsma K.A., Hendrickx F., Grbić V., van Leeuwen T. (2015). Adaptation of a polyphagous herbivore to a novel host plant extensively shapes the transcriptome of herbivore and host. Mol. Ecol..

[32] Díaz-Riquelme J., Zhurov V., Rioja C., Pérez-Moreno P., Badja S., van Leeuwen T., Martínez-Zapater J.M., Grbic M., Grbic V. (2016). Comparative genome-wide transcriptome analysis of *Vitis vinifera* responses to adapted and non-adapted strains of two-spotted spider mite, *Tetranuchus urticae*. BMC Genomics.

[33] Santamaría M.E., González-Cabrera J., Martínez M., Grbic V., Castañera P., Díaz I., Ortego F. (2015). Digestive proteases in bodies and faeces of the two-spotted spider mite, *Tetranychus urticae*. J. Insect. Phys..

[34] Hussey N.W., Parr W.J. (1963). Dispersal of the glasshouse red spider mite *Tetranychus urticae* Koch (Acarina, Tetranychidae). Entomol. Exp. Appl..

[35] Li J., Margolies D.C. (1993). Effects of mite age, mite density, and host quality on aerial dispersal behavior in the two spotted spider mite. Entomol. Exp. Appl..

[36] Osakabe M.H., Isobe H., Kasai A., Masuda R., Kubota S., Umeda M. (2008). Aerodynamic advantages of upside down take-off for aerial dispersal in *Tetranychus* spider mites. Exp. Appl. Acarol..

[37] Clotuche G., Mailleux A.C., Fernández A.A., Deneubourg J.L., Detrain C., Hance T. (2011). The formation of collective silk balls in the spider mite *Tetranychus urticae* Koch. PLoS ONE.

[38] Knowles C.O. (1997). Mechanisms of resistance to acaricides. Molecular Mechanisms of Resistance to Agrochemicals.

[39] Van Leeuwen T., Vanholme B., van Pottelberge S., van Nieuwenhuyse P., Nauen R., Tirry L., Denholm I. (2008). Mitochondrial heteroplasmy and the evolution of insecticide resistance: Non-Mendelian inheritance in action. Proc. Natl. Acad. Sci. USA.

[40] (2017). Michigan State University. https://www.pesticideresistance.org/search.php.

[41] IRAC (2017). IRAC MoA Classification Scheme. Version 8.3. http://www.irac-online.org/documents/moa-classification/?ext=pdf.

[42] Rabinge R., Helle W., Sabelis M.W. (1985). Aspects of damage assessment. Spider Mites. Their Biology, Natural Enemies and Control.

[43] Marcic D. (2012). Acaricides in modern management of plant-feeding mites. J. Pest Sci..

[44] Van Leeuven T., Dermauw W., Grbic M., Tirry L., Feyereisen R. (2013). Spider mite control and resistance management: Does a genome help?. Pest Manag. Sci..

[45] Dekeyser M A. (2005). Acaricide mode of action. Pest Manag. Sci..

[46] Pascual-Ruiz S., Aguilar-Fenollosa E., Ibáñez-Gual V., Hurtado-Ruiz M.A., Martínez-Ferrer M.T., Jacas J.A. (2014). Economic threshold for *Tetranychus urticae* (Acari: Tetranychidae) in clementine mandarins *Citrus clementina*. Exp. Appl. Acarol..

[47] García-Marí F., González-Zamora J.E. (1999). Biological control of *Tetranychus urticae* (Acari: Tetranychidae) with naturally occurring predators in strawberry plantings in Valenica, Spain. Exp. Appl. Acarol..

[48] Eilenberg J., Hajek A., Lomer C. (2001). Suggestions for unifying the terminology in biological control. BioControl.

[49] De Moraes G.J., McMurtry J.A., Denmark H.A., Cambos C.B. (2004). A revised catalog of the mite family *Phytoseiidae*. Zootaxa.

[50] Mcmurtry J.A., de Moraes G.J., Sourassou N.F. (2013). Revision of the lifestyles of phytoseiid mites (Acari: Phytoseiidae) and implications for biological control strategies. Syst. Appl. Acarol..

[51] Croft B.A., Brown A.W.A. (1975). Responses of arthropod natural enemies to insecticides. Annu. Rev. Entomol..

[52] Stark J.D., Vargas R., Banks J.E. (2007). Incorporating ecologically relevant measures of pesticide effect for estimating the compatibility of pesticides and biocontrol agents. J. Econ. Entomol..

[53] Tang J., Li J., Shao Y., Yang B., Liu Z. (2010). Fipronil resistance in the whitebacked planthopper (*Sogatella furcifera*): Possible resistance mechanisms and cross-resistance. Pest Manag. Sci..

[54] Stark J.D., Banks J.E. (2003). Population-level effects of pesticides and other toxicants on arthropods. Annu. Rev. Entomol..

[55] Desneux N., Decourtye A., Delpuech J.M. (2007). The sublethal effects of pesticides on beneficial arthropods. Annu. Rev. Entomol..

[56] Cloyd R.A. (2012). Indirect Effects of Pesticides on Natural Enemies. Pesticides—Advances in Chemical and Botanical Pesticides.

[57] Longley M., Jepson P.C. (1996). Effects of honeydew and insecticide residues on the distribution of foraging aphid parasitoids under glasshouse and field conditions. Entomol. Exp. Appl..

[58] Desneux N., Rafalimanana H., Kaiser L. (2004). Dose–response relationship in lethal and behavioural effects of different insecticides on the parasitic wasp *Aphidius ervi*. Chemosphere.

[59] Costa Comelles J., Bosch Serra À.D., Botargues A., Cabiscol P., Moreno A., Portillo J., Avilla Hernández J. (1997). Acción de algunos acaricidas sobre los fítoseídos y la araña roja *Panonychus ulmi* (Koch) en manzano. Boletín de Sanidad Vegetal. Plagas.

[60] Stanyard M.J., Foster R.E., Gibb T.J. (1998). Population dynamics of *Amblyseius fallacies* (Acari: Phytoseiidae) and european red mite (Acari: Tetranychidae) in apple trees treated with selected acaricides. J. Econ. Entomol..

[61] Guedes R.N.C., Magalhaes L.C., Cosme L.V. (2009). Stimulatory sublethal response of a generalist predator to permethrin: Hormesis, hormoligosis, or homeostatic regulation?. J. Econ. Entomol..

[62] James D.G., Price T.S. (2002). Fecundity in two spotted spider mite (Acari: Tetranychidae) is increased by direct and systemic exposure to imidacloprid. J. Econ. Entomol..

[63] Marcic D. (2003). The effects of clofentezine on life-table parameters in two-spotted spider mite *Tetranychus urticae*. Exp. Appl. Acarol..

[64] Li X., Garvey M.K.I., Li B., Carrillo J. (2017). Domestication of tomato has reduced the attraction of herbivore natural enemies to pest-damaged plants. Agric. For. Entomol..

[65] Brummer C.E., Barber W.T., Collier S.M., Cox T.S., Johnson R., Murray S.C., Olsen R.T., Pratt R.C., Thro A.M. (2011). Plant breeding for harmony between agriculture and the environment. Front. Ecol. Environ..

[66] Carmona D., Lajeunesse M.J., Johnson M.T. (2011). Plant traits that predict resistance to herbivores. Funct. Ecol..

[67] Stowe K.A., Marquis R.J., Hochwender C.G., Simms E.L. (2000). The evolutionary ecology of tolerance to consumer damage. Annu. Rev. Ecol. Syst..

[68] Schaller A., Howe G.A., Schaller A. (2008). Direct Defenses in Plants and Their Induction by Wounding and Insect Herbivores. Induced Plant Resistance to Herbivory.

[69] Wittstock U., Gershenzon J. (2002). Constitutive plant toxins and their role in defense against herbivores and pathogens. Curr. Opin. Plant Biol..

[70] Huchelmann A., Boutry M., Hachez C. (2017). Plant glandular trichomes: Natural cell factories of high biotechnological interest. Plant Physiol..

[71] Tian D., Tooker J., Peiffer M., Chung S.H., Felton G.W. (2012). Role of trichomes in defense against herbivores: comparison of herbivore response to woolly and hairless trichome mutants in tomato (*Solanum lycopersicum*). Planta.

[72] Karley A. J., Mitchell C., Brookes C., McNicol J., O’Neill T., Roberts H., Graham J., Johnson S.N. (2016). Exploiting physical defence traits for crop protection: leaf trichomes of *Rubus idaeus* have deterrent effects on spider mites but not aphids. Annu. Appl. Biol..

[73] Eigenbrode S.D. (2004). The effects of plant epicuticular waxy blooms on attachment and effectiveness of predatory insects. Arthropod. Struct. Dev..

[74] VanEtten H.D., Mansfield J.W., Bailey J.A., Farmer E.E. (1994). Two classes of plant antibiotics: Phytoalexins versus “phytoanticipins”. Plant Cell.

[75] Stauber E.J., Kuczka P., van Ohlen M., Vogt B., Janowitz T., Piotrowski M., Beuerle T., Wittstock U. (2012). Turning the “mustard oil bomb” into a “cyanide bomb”: Aromatic glucosinolate metabolism in a specialist insect herbivore. PLoS ONE.

[76] Miresmailli S., Isman M.B. (2014). Botanical insecticides inspired by plant–herbivore chemical interactions. Trends Plant Sci..

[77] Rioja C., Zhurov V., Bruinsma K., Grbic M., Grbic V. (2017). Plant-herbivore interactions: A case of an extreme generalist, the two-spotted spider mite *Tetranychus urticae*. MPMI.

[78] Alba J.M., Schimmel B.C.J., Glas J.J., Ataide L.M.S., Pappas M.L., Villarroel C.A., Schuurink R.C., Sabbelis M.W., Kant M.R. (2015). Spider mites suppress tomato defenses downstream of jasmonate and salicylate independently of hormonal crosstalk. New Phytol..

[79] Sarmento R.A., Lemos F., Bleeker P.M., Schuurink R.C., Pallini A., Oliveira M.G.A., Lima E.R., Kant M., Sabelis M.W., Janssen A. (2011). A herbivore that manipulates plant defence. Ecol. Lett..

[80] Pieterse C.M., Leon-Reyes A., van der Ent S., van Wees S.C. (2009). Networking by small-molecule hormones in plant immunity. Nat. Chem. Biol..

[81] Villarroel C.A., Jonckheere W., Alba J.M., Glas J.J., Dermauw W., Haring M.A., van Leeuwen T., Schuurink R.C., Kant M.R. (2016). Salivary proteins of spider mites suppress defenses in *Nicotiana benthamiana* and promote mite reproduction. Plant J..

[82] Bensoussan N., Santamaria M.E., Zhurov V., Diaz J., Grbić M., Grbić V. (2016). Plant–herbivore interaction: dissection of the cellular pattern of *Tetranychus urticae* feeding on the host plant. Front. Plant Sci..

[83] Jones J.D.G., Dangl J.L. (2006). The plant immune system. Nature.

[84] Mithöfer A., Boland W. (2008). Recognition of herbivory-Associated Molecular Patterns. Plant Phys..

[85] Arimura G.I., Ozawa R., Nishioka T., Boland W., Koch T., Kühnemann F., Takabayashi J. (2002). Herbivore-induced volatiles induce the emission of ethylene in neighboring lima bean plants. Plant J..

[86] Leitner M., Boland W., Mithöfer A. (2005). Direct and indirect defenses induced by piercing-sucking and chewing herbivores in *Medicago truncatula*. New Phytol..

[87] Kant M.R., Ament K., Sabelis M.W., Haring M.A., Schuurink R.C. (2004). Differential timing of spider mite-induced direct and indirect defenses in tomato plants. Plant Phys..

[88] Kawazu K., Mochizuki A., Sato Y., Sugeno W., Murata M., Seo S., Mitsuhara I. (2012). Different expression profiles of jasmonic acid and salicylic acid inducible genes in the tomato plant against herbivores with various feeding modes. Arthropod-Plant Interact..

[89] Zhurov V., Navarro M., Bruinsma K.A., Arbona V., Santamaria M.E., Cazaux M., Wybouw N., Osborne E.J., Ens C., Rioja C. (2014). Reciprocal responses in the interaction between *Arabidopsis* and the cell-content-feeding chelicerate herbivore spider mite. Plant Physiol..

[90] Maserti B.E., del Carratore R., Della Croce C.M. (2011). Comparative analysis of proteome changes induced by the two-spotted spider mite *Tetranychus urticae* and methyl jasmonate in citrus leaves. J. Plant Phys..

[91] Agut B., Gamir J., Jacas J.A., Hurtado M., Flors V. (2014). Different metabolic and genetic responses in citrus may explain relative susceptibility to *Tetranychus urticae*. Pest Manag. Sci..

[92] Kant M.R., Sabelis M.W., Haring M.A., Schuurink R.C. (2008). Intraspecific variation in a generalist herbivore accounts for differential induction and impact of host plant defenses. Proc. R Soc. B: Biol. Sci..

[93] Jonckheere W., Dermauw W., Khalighi M., Pavlidi N., Reubens W., Baggerman G., Tirry L., Menschaert G., Kant M.R., Vanholeme B. (2018). A gene family coding for salivary proteins (SHOT) of the polyphagous spider mite *Tetranychus urticae* exhibits fast host-dependent transcriptional plasticity. MPMI.

[94] Glas J.J., Alba J.M., Simoni S., Villarroel C.A., Stoops M., Schimmel B.C.J., Schuurink R.C., Sabelis M.W., Kant M.R. (2014). Defense suppression benefits herbivores that have a monopoly on their feeding site but can backfire within natural communities. BMC Biol..

[95] Castañé C., Arnó J., Gabarra R., Alomar O. (2011). Plant damage to vegetable crops by zoophytophagus mirid predators. Biol. Control.

[96] Pappas M.L., Steppuhn A., Geuss D., Topalidou N., Aografou A., Sabelis M.W., Broufas G.D. (2015). Beyond predation: The zoophytophagous predator macrolophus pygmaeus induces tomato resistance against spider mites. PLoS ONE.

[97] Zhang P.J., Zheng S.J., van Loon J.J., Boland W., David A., Mumm R., Dicke M. (2009). Whiteflies interfere with indirect plant defense against spider mites in Lima bean. Proc. Nat. Acad. Sci. USA.

[98] Bleeker P.M., Mirabella R., Diergaarde P.J., VanDoorn A., Tissier A., Kant M.R., Schuurink R.C. (2012). Improved herbivore resistance in cultivated tomato with the sesquiterpene biosynthetic pathway from a wild relative. Proc. Nat. Acad. Sci. USA.

[99] Cavalcanti S.C.H., Niculau E.D.S., Blank A.F., Câmara C.A.G., Araújo I.N., Alves P.B. (2010). Composition and acaricidal activity of *Lippia sidoides* essential oil against two-spotted spider mite (*Tetranychus urticae* Koch). Bioresour Technol..

[100] Bleeker P.M., Diergaarde P.J., Ament K., Schütz S., Johne B., Dijkink J., Hiemstra H., de Gelder R., de Both M.T.J., Sabelis M.W. (2011). Tomato-produced 7-epizingiberene and R-curcumene act as repellents to whiteflies. Phytochemistry.

[101] Ahmad S., Gordon-Weeks R.U.T.H., Pickett J., Ton J. (2010). Natural variation in priming of basal resistance: from evolutionary origin to agricultural exploitation. Mol. Plant Pathol..

[102] Schlaeppi K., Abou-Mansour E., Buchala A., Mauch F. (2010). Disease resistance of Arabidopsis to Phytophthora brassicae is established by the sequential action of indole glucosinolates and camalexin. Plant J..

[103] Winde I., Wittstock U. (2011). Insect herbivore counteradaptations to the plant glucosinolate-myrosinase system. Phytochemistry.

[104] Badenes-Perez F.R., Reichelt M., Gershenzon J., Heckel D.G. (2013). Interaction of glucosinolate content of *Arabidopsis thaliana* mutant lines and feeding and oviposition by generalist and specialist lepidopterans. Phytochemistry.

[105] Santamaria M.E., Martínez M., Arnaiz A., Ortego F., Grbic V., Diaz I. (2017). MATI, a novel protein involved in the regulation of herbivore-associated signaling pathways. Front. Plant Sci..

[106] Pourcel L., Irani N.G., Koo A.J., Bohorquez-Restrepo A., Howe G.A., Grotewold E. (2013). A chemical complementation approach reveals genes and interactions of flavonoids with other pathways. Plant J..

[107] Onkokesung N., Reichelt M., van Doorn A., Schuurink R.C., van Loon J.J., Dicke M. (2014). Modulation of flavonoid metabolites in Arabidopsis thaliana through overexpression of the MYB75 transcription factor: role of kaempferol-3, 7-dirhamnoside in resistance to the specialist insect herbivore *Pieris brassicae*. J. Exp. Bot..

[108] Agut B., Gamir J., Jaques J.A., Flors V. (2015). *Tetranychus urtichae*-triggered responses promote genotype-dependent conspecific repellence or attractiveness in citrus. New Phyt..

[109] Mauch-Mani B., Bacelli I., Luna E., Flors V. (2017). Defense priming: An adaptative part of induced resistance. Annu. Rev. Plant. Biol..

[110] Martínez-Medina A., Flors V., Heil M., Mauch-Mani B., Pieterse C.M., Pozo M.J., Ton J., van Dam N. M., Conrath U. (2016). Recognizing plant defense priming. Trends Plant Sci..

[111] Alba J.M., Montserrat M., Fernández-Muñoz R. (2009). Resistance to the two-spotted spider mite (*Tetranychus urticae*) by acylsucroses of wild tomato (*Solanum pimpinellifolium*) trichomes studied in a recombinant inbred line population. Exp. Appl. Acarol..

[112] Hare J.D., Walling L.L. (2006). Constitutive and jasmonate-inducible traits of *Datura wrightii*. J. Chem. Ecol..

[113] Green T.R., Ryan C.A. (1972). Wound-induced proteinase inhibitor in plant leaves: a possible defense mechanism against insects. Science.

[114] Ryan C.A. (1990). Protease inhibitors in plants: Genes for improving defenses against insects and pathogens. Annu. Rev. Phytopathol..

[115] Lison P., Rodrigo I., Conejero V. (2006). A novel function for the cathepsin D inhibitor in tomato. Plant Phys..

[116] Zavala J.A., Patankar A.G., Gase K., Hui D.Q., Baldwin I.T. (2004). Manipulation of endogenous trypsin proteinase inhibitor production in *Nicotiana attenuata* demonstrates their function as antiherbivore defenses. Plant Phys..

[117] Talyzina N.M., Ingvarsson P.K. (2006). Molecular evolution of a small gene family of wound inducible *Kunitz trypsin* inhibitors in *Populus*. J. Mol. Evol..

[118] Carrillo L., Martinez M., Álvarez-Alfageme F., Castañera P., Smagghe G., Diaz I., Ortego F. (2011). A barley cysteine-proteinase inhibitor reduces the performance of two aphid species in artificial diets and transgenic Arabidopsis plants. Transgenic Res.

[119] Santamaria M.E., Cambra I., Martinez M., Pozancos C., Gonzalez-Melendi P., Grbic V., Castañera P., Ortego F., Diaz I. (2012). Gene pyramiding of peptidase inhibitors enhances plant resistance to the spider mite *Tetranychus urticae*. PLoS ONE.

[120] Pieterse C.M., Zamioudis C., Berendsen R.L., Weller D.M., van Wees S.C., Bakker P.A. (2014). Induced Systemic Resistance by Beneficial Microbes. Annu. Rev. Phytopathol..

[121] Erb M., Köllner T.G., Degenhardt J., Zwahlen C., Hibbard B.E., Turlings T.C. (2011). The role of abscisic acid and water stress in root herbivore-induced leaf resistance. New Phytologist.

[122] Karban R., Carey J.R. (1984). Induced resistance of cotton seedlings to mites. Science.

[123] Mousavi S.A., Chauvin A., Pascaud F., Kellenberger S., Farmer E.E. (2013). *GLUTAMATE RECEPTOR-LIKE* genes mediate leaf-to-leaf wound signalling. Nature.

[124] Agut B., Gamir J., Jaques J.A., FLors V. (2016). Systemic resistance in citrus to *Tetranychus urticae* induced by conspecifics is transmitted by grafting mediated by mobile amino acids. J. Exp. Bot..

[125] Dicke M. (1986). Volatile spider-mite pheromone and host-plant kairomone, involved in spaced-out gregariousness in the spider mite *Tetranychus urticae*. Physiol. Entomol..

[126] Pallini A., Janssen A., Sabelis M.W. (1997). Odour-mediated responses of phytophagous mites to conspecific and heterospecific competitors. Oecologia.

[127] Frost C.J., Mescher M.C., Carlson J.E., De Moraes C.M. (2008). Plant defense priming against herbivores: getting ready for a different battle. Plant Physiol..

[128] Baldwin I., Schultz J.C. (1983). Talking trees. Science.

[129] Engelberth J., Alborn H.T., Schmelz E.A., Tumlinson J.H. (2004). Airborne signals prime plants against insect herbivore attack. Proc. Nat. Acad. Sci. USA.

[130] Muroi A., Ramadan A., Nishihara M., Yamamoto M., Ozawa R., Takabayashi J., Arimura G.I. (2011). The composite effect of transgenic plant volatiles for acquired immunity to herbivory caused by inter-plant communications. PLoS ONE.

[131] Aguilar-Fenollosa E., Pina T., Gómez-Martínez M.A., Hurtado M.A., Jacas J.A. (2012). Does host adaptation of *Tetranychus urticae* populations in clementine orchards with a *Festuca arundinacea* cover contribute to a better natural regulation of this pest mite?. Entomol. Exp. App..

[132] Van Wijk M., de Bruijn P.J., Sabelis M.W. (2008). Predatory mite attraction to herbivore-induced plant odors is not a consequence of attraction to individual herbivore-induced plant volatiles. J. Chem. Ecol..

[133] Aguilar-Fenollosa E., Ibáñez-Gual M.V., Pascual-Ruiz S., Hurtado M., Jacas J.A. (2011). Effect of ground-cover management on spider mites and their phytoseiid natural enemies in clementine mandarin orchards (I): Bottom-up regulation mechanisms. Biol. Control.

[134] De Boer J.G., Dicke M. (2004). The role of methyl salicylate in prey searching behavior of the predatory mite *Phytoseiulus persimilis*. J. Chem. Ecol..

[135] Ament K., Krasikov V., Allmann S., Rep M., Takken F.L., Schuurink R.C. (2010). Methyl salicylate production in tomato affects biotic interactions. Plant J..

[136] Kappers I.F., Hoogerbrugge H., Bouwmeester H.J., Dicke M. (2011). Variation in herbivory-induced volatiles among cucumber (*Cucumis sativus* L.) varieties has consequences for the attraction of carnivorous natural enemies. J. Chem. Ecol..

[137] Brillada C., Nishihara M., Shimoda T., Garms S., Boland W., Maffei M.E., Arimura G.I. (2013). Metabolic engineering of the C16 homoterpene TMTT in Lotus japonicus through overexpression of (*E*,*E*)-geranyllinalool synthase attracts generalist and specialist predators in different manners. New Phytol..

[138] Pozo M.J., Jung S.C., Martínez-Medina A., López-Ráez J.A., Azcón-Aguilar C., Barea J.M. (2013). Root allies: Arbuscular mycorrhizal fungi help plants to cope with biotic stresses. Symbiotic Endophytes.

[139] Schausberger P., Peneder S., Juerschik S., Hoffmann D. (2012). Mycorrhiza changes plant volatiles to attract spider mite enemies. Func. Ecol..

[140] Rashid M.H., Chung Y.R. (2017). Induction of systemic resistance against insect herbivores in plants by beneficial soil microbes. Front. Plant Sci..

[141] Sharma E., Anand G., Kapoor R. (2017). Terpenoids in plant and arbuscular mycorrhiza-reinforced defence against herbivorous attack. Ann. Bot..

[142] Mérey G.V., Veyrat N., Mahuku G., Valdez R.L., Turlings T.C., D’Alessandro M. (2011). Dispensing synthetic green leaf volatiles in maize fields increases the release of sesquiterpenes by the plants, but has little effect on the attraction of pest and beneficial insects. Phytochemistry.

[143] Uefune M., Choh Y., Abe J., Shiojiri K., Sano K., Takabayashi J. (2012). Application of synthetic herbivore-induced plant volatiles causes increased parasitism of herbivores in the field. J. App. Entomol..

[144] Sun X.L., Wang G.C., Gao Y., Chen Z.M. (2012). Screening and field evaluation of synthetic volatile blends attractive to adults of the tea weevil, *Myllocerinus aurolineatus*. Chemoecology.

[145] Simpson M., Gurr G.M., Simmons A.T., Wratten S.D., James D.G., Leeson G., Nicol H.I. (2011). Insect attraction to synthetic herbivore-induced plant volatile-treated field crops. Agr. For. Entomol..

[146] Rodriguez-Saona C., Kaplan I., Braasch J., Chinnasamy D., Williams L. (2011). Field responses of predaceous arthropods to methyl salicylate: A meta-analysis and case study in cranberries. Biol. Control.

[147] Adar E., Inbar M., Gal S., Gan-Mor S., Palevsky E. (2014). Pollen on-twine for food provisioning and oviposition of predatory mites in protected crops. BioControl.

[148] Aguilar-Fenollosa E., Jacas J.A. (2013). Effect of ground cover management on *Thysanoptera* (thrips) in clementine mandarin orchards. J. Pest Sci..

[149] Pina T., Argolo P.S., Urbaneja A., Jacas J.A. (2012). Effect of pollen quality on the efficacy of two different life-style predatory mites against Tetranychus urticae in citrus. Biol. Control.

[150] Gómez-Martínez M.A., Aguilar-Fenollosa E., Jaques J.A., Pina T. (2018). Ecobiology of *Anaphothrips obscurus*, a new dweller of citrus orchards brought in by more sustainable pest management practices. Agric. For. Entomol..

[151] Jaques J.A., Aguilar-Fenollosa E., Hurtado M.A., Pina M.T., Carrillo D., de Moraes G.J., Peña J.E. (2015). Food web engineering to enhance biological control of *Tetranychus urticae* by phytoseiid mites (Tetranychidae: Phytoseiidae) in Citrus. Prospects for Biological Control of Plant Feeding Mites and Other Harmful Organisms. Progress in Biological Control.

